# Laminin chain assembly is regulated by specific coiled-coil interactions

**DOI:** 10.1016/j.jsb.2010.02.004

**Published:** 2010-05

**Authors:** Philip R. Macdonald, Ariel Lustig, Michel O. Steinmetz, Richard A. Kammerer

**Affiliations:** a Wellcome Trust Centre for Cell-Matrix Research, Faculty of Life Sciences, University of Manchester, Manchester M13 9PT, UK; b Structural Biology, Biozentrum, University of Basel, CH-4056 Basel, Switzerland; c Biomolecular Research, Structural Biology, Paul Scherrer Insititut, CH-5232 Villigen PSI, Switzerland

**Keywords:** AUC, CD spectroscopy, Coiled coil, EM, Laminin, Non-denaturing PAGE

## Abstract

Laminins are large heterotrimeric, multidomain proteins that play a central role in organising and establishing all basement membranes. Despite a total of 45 potential heterotrimeric chain combinations formed through the coiled-coil domain of the 11 identified laminin chains (α1–5, β1–3, γ1–3), to date only 15 different laminin isoforms have been reported. This observation raises the question whether laminin assembly is regulated by differential gene expression or specific chain recognition. To address this issue, we here perform a complete analysis of laminin chain assembly and specificity. Using biochemical and biophysical techniques, all possible heterotrimeric combinations from recombinant C-terminal coiled-coil fragments of all chains were analysed. Apart from laminin 323 (α3, β2, γ3), for which no biochemical evidence of its existence *in vivo* is available, these experiments confirmed all other known laminin isoforms and identified two novel potential chain combinations, laminins 312 (α3, β1, γ2) and 422 (α4, β2, γ4). Our findings contribute to the understanding of basement membrane structure, function and diversity.

## 1 Introduction

Laminins are multidomain proteins found mainly in basement membranes, where they function as structural components that provide an integral part of the basement membrane scaffolding (Miner, 2008; Yurchenco and Patton, 2009). In fact, in the absence of collagen IV, the other major component that assembles into a scaffold, laminin has been shown to be sufficient for forming basement membrane-like matrices during early development of mice embryos (Poschl et al., 2004). In addition to this structural role, laminins also interact with cell surface receptors, such as intergins, which control signalling events that regulate cell proliferation, cell migration, cell differentiation and cell morphology (Miner and Yurchenco, 2004).

Laminins are large (400–600 kDa) heterotrimeric glycoproteins that contain one copy each of an α, β and γ chain (Miner, 2008). Structural analysis using electron microscopy revealed laminin as a cruciform-like structure with three short arms and one long arm (Engel et al., 1981). The long arm consists of an α-helical coiled coil and is the only region in which all three chains associate (Paulsson et al., 1985). Since the first laminin was isolated in 1979 from the mouse Engelbreth–Holm–Swarm (EHS) tumour (Timpl et al., 1979), 11 distinct laminin genes (α1–5, β1–3, γ1–3) have been identified (Miner, 2008; Miner and Yurchenco, 2004). During development and in adult organisms, different cell and tissue types each have their own specific and temporally regulated laminin expression, often producing more than one isoform (Miner, 2008; Miner and Yurchenco, 2004). For example, α chains are expressed in overlapping but distinct patterns, with each basement membrane containing at least one of the known α chains (Miner et al., 1997). The existence of many laminin isoforms with overlapping expression patterns suggests specific chain recognition. Previous investigations have highlighted the high selectivity and specificity within the laminin coiled-coil assembly domain, demonstrating that the extreme C-terminal fragment of the coiled-coil domain contains the chain recognition sites and has the same oligomerisation state and a similar stability as the native full-length laminin protein. In previous experiments (Paulsson et al., 1985; Hunter et al., 1990, 1992), the selective recognition of the laminin chains α1, β1, and γ1 was studied using the elastase-derived E8 fragment of laminin 111. This fragment comprises approximately the C-terminal half of the coiled-coil domain and showed that after complete denaturation with urea the chains could be renatured to produce a single, highly specific product, which was indistinguishable from the native E8 fragment. Further evidence for the C-terminus of the coiled-coil domain providing the laminin isoform chain specificity was demonstrated by recombinant laminin 111 chain fragments based on the native 25 K fragment or by synthetic peptides representing the C-terminal 50 amino acid region of the long arm (Nomizu et al., 1992, 1994; Kammerer et al., 1995). In the absence of α1 chains, β1γ1 heterodimers were preferred over homoassociations; however, addition of the α chain resulted in heterotrimer formation accompanied with a increase in thermal stability and was preferred over any other chain combination. Assembly models have suggested that a βγ heterodimer is initially formed with the α chain interacting as a secondary event, allowing for the formation of a more stable three-stranded coiled-coil structure (Kammerer et al., 1995; Nomizu et al., 1996). It has been postulated that specific regions within the coiled-coil domain are responsible for the correct assembly and folding of laminins (Utani et al., 1994, 1995). Deletion mapping in the β1 and γ1 chains identified two sites important for heterodimerization and heterotrimerization, and in the α1 and α2 chains one critical site for trimerization was identified. Site-directed mutagenesis revealed that charged amino acid residues within these sites were essential for association. However, recombinant laminin β1 and γ1 chain fragments based on the native 25 K fragment, in which the proposed assembly region critical for dimer formation was missing in γ1, still showed association into a stable β1γ1 complex (Kammerer et al., 1995).

There are a total 45 different possible heterotrimeric laminin combinations, of which only 15 isoforms have been identified *in vivo* to date (Miner, 2008). The molecular basis for this high selectivity of chain association is unclear. To address this open issue, we here perform a complete analysis of laminin chain assembly and specificity, using recombinant C-terminal coiled-coil fragments corresponding to the E8 fragment of each laminin chain.

## 2 Materials and methods

### 2.1 Construction of expression plasmids

The cDNA clones for mouse laminin chains α1, α2, β1, β3, γ1, and γ2 were provided by Dr. Y. Yamada, National Institutes of Health, Bethesda, Maryland, USA. The cDNA clones for the laminin α3 and α5 chain were made available by Dr. D. Aberdam, INSERM U898, Nice, France and Dr. J.H. Miner, Washington University School of Medicine, St. Louis, USA, respectively. The remaining laminin chain cDNA clones were obtained from Geneservice (UK). All cDNA clones were used as templates in PCR reactions to produce DNA fragments encoding the mouse C-terminal coiled-coil laminin chain fragments shown in Table 1. The amplified PCR products were ligated into bacterial expression vectors pPEP-T (Brandenberger et al., 1996) (α1–4, γ1 and γ2 chains) or pHis (Kammerer et al., 1998) (α5, β1–3 and γ3 chains), which is a modified version of the pET-15b vector (Merck Biosciences). Both vectors encode a 6xHis tag and a thrombin cleavage site immediately preceding the laminin sequences. Recombinant plasmid DNA was purified from JM109 *Escherichia coli* cloning host strain using a QIAprep spin plasmid kit (QIAGEN) and recombinant insert DNA was verified by DNA sequencing.

### 2.2 Expression and purification of recombinant proteins

The recombinant laminin chains were expressed in *E. coli* JM109(DE3) host strain (Merck Biosciences) as previously described (Kammerer et al., 1995). The 6xHis-tagged proteins were purified by immobilized metal affinity chromatography on Ni^2+^-Sepharose (GE Healthcare). Purification was performed under denaturing condition using 8 M urea as described in the pET system manual (Merck Biosciences). Removal of the 6xHis tags by thrombin was performed as previously described (Kammerer et al., 1995). Two residues, GlySer, which were derived from the protease recognition site, remained at the N termini of the fragments not belonging to the laminin sequences. Determination of protein concentration was achieved by the absorbance of tryptophan and tyrosine residues at 280 nm (Edelhoch, 1967).

### 2.3 Gel electrophoresis and protein complex identification

SDS–PAGE was performed using 15% gels and non-denaturing PAGE was performed using 10% gels. For non-denaturing PAGE of complexes with a predicted overall negative net charge, the standard Laemmli gel system without SDS was used (Hunter et al., 1992, 1990). For basic protein complexes, acid gels were used as described on the webpage of The Protein Purification Facility, The Wolfson Centre for Applied Structural Biology, The Hebrew University of Jerusalem, Israel. Before loading samples for non-denaturing gels, protein mixtures were reduced and heated to 70 °C for 5 min and allowed to cool. All non-denaturing gels were run overnight at 50 V. All protein bands on gels were visualised by Coomassie blue staining.

To confirm their chain composition, complexes were excised from non-denaturing polyacrylamide gels. Gel slices were thoroughly washed four times with 200 μl of milliQwater. They were then dehydrated using subsequent 10-min washes of 200 μl of 50% acetonitrile, 100 μl of 50 mM ammonium bicarbonate and 200 μl of 50% acetonitrile. Re-swelling of gel slices and digestion of the proteins with trypsin was achieved by adding 20 μl of 50 mM ammonium hydrogen carbonateand 1 μl of trypsin (Sigma; 1 μg/μl stock), followed by overnight incubation at 37 °C. Supernatants were removed and retained, and digested peptides were extracted from the gel slice by three cycles of incubation with 20 μl of 70% acetonitrile for 15 min. All supernatants were pooled and the sample volume was reduced using a Hetovac (Heto-Holten) and adjusted to 20 μl containing 0.1% formic acid. Q-TOF mass spectrometry was performed by the Biomolecular Analysis Core Facility, University of Manchester, UK, and resulting masses were used to match proteins by peptide mass fingerprinting.

### 2.4 Circular dichroism (CD) spectroscopy

All CD studies were carried out using a Jasco J-810 spectropolarimeter fitted with a single cell Peltier temperature controller. All proteins were in PBS (5 mM sodium phosphate, pH 7.4, 150 mM NaCl). A cuvette of 0.1 cm pathlength was used. Data points were recorded at 1-nm intervals using a 2-nm bandwidth and 16-s response times.

Temperature dependencies at 222 nm of the CD signal and spectra were measured starting at 4 °C using a ramping rate of 1 °C/min and a 1 nm bandwidth. In all experiments, baseline signals for buffer alone were measured and subtracted from the raw data.

### 2.5 Analytical ultracentrifugation (AUC)

Sedimentation equilibrium and sedimentation velocity experiments were performed on a Beckman Optima XL-A analytical ultracentrifuge equipped with 12-mm Epon double-sector cells in an An-60 Ti rotor. The laminin complexes were analysed in 5 mM sodium phosphate buffer (pH 7.0) supplemented with 150 mM sodium chloride, and protein concentrations were adjusted to 0.1–0.5 mg/mL. Sedimentation velocity runs were performed at 20 °C at a rotor speed of 52,000 rpm and a protein concentration of 0.5 mg/ml, and sedimenting material was assayed by its absorbance at 234 or 278 nm. Sedimentation coefficients were corrected to standard conditions (water, 20 °C). Sedimentation equilibrium scans were carried out at four different protein concentrations ranging from 0.1 to 0.5 mg/ml and rotor speeds of 13,000–24,000 rpm, depending on molecular mass. Average molecular masses were evaluated by using a floating baseline computer program to obtain the best linear fit of ln*A* versus *r*^2^ where *A* is the absorbance and *r* the distance from the rotor centre. A partial specific volume of 0.73 ml/g was used for all calculations.

### 2.6 Electron microscopy

For glycerol spraying/low-angle rotary metal-shadowing, 20 μl protein samples, 0.1–0.3 mg/ml in PBS, 30% glycerol, were sprayed onto freshly cleaved mica at room temperature and rotary shadowed in a BA 511 M freeze-etch apparatus (Balzers) with platinum/carbon at an elevation angle of 3–5° (Fowler and Aebi, 1983). Electron micrographs were taken in a Philips Morgagni TEM operated at 80 kV equipped with a Megaview III CCD camera at 54,000× nominal magnification.

## 3 Results and discussion

### 3.1 Design rationale and expression of laminin coiled-coil fragments

Previous studies have demonstrated that laminin assembly starts from the C-terminal end of the coiled-coil domain (Paulsson et al., 1985; Hunter et al., 1990, 1992; Kammerer et al., 1995; Nomizu et al., 1992, 1994, 1996; Utani et al., 1995, 1994). Accordingly, laminin C-terminal coiled-coil fragments ranging from 50 to 200 amino acids in length assemble into heterotrimeric structures with a similar stability, compared to native laminin. For the purpose of this study, all recombinant chain fragments were based on the E8 fragment of laminin 111. The E8 fragment consists of the C-terminal half the coiled-coil domain which can be denaturated and reassembled into molecules having an α-helix content, apparent molecular mass, chain composition and ultrastructural appearance that is indistinguishable from the native fragment (Paulsson et al., 1985; Hunter et al., 1990, 1992). In order to produce the α chain fragments of the present study, the crystal structure of the LG5 domain of the laminin α2 chain from mouse (Hohenester et al., 1999) was used as a basis to identify the C-terminal end of each coiled-coil domain. In β and γ chains, the end of the amino-acid sequence naturally corresponds to the C-terminus of the coiled-coil domain. The N-terminal sequences of α1, β1 and γ1 from the E8 fragment were used to identify the equivalent N-terminal amino acid positions in the remaining laminin chains (Deutzmann et al., 1988). The C-terminal mouse α, β and γ chain fragments range in size from 215 to 246 residues. Details regarding the length of individual chain fragments and their overall net charge are given in Table 1.

**Fig. 1:**
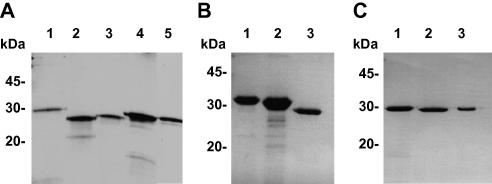
Purified recombinant laminin coiled-coil fragments. SDS–PAGE analysis of individual chains under reducing conditions. (A) Lane 1, α1; lane 2, α2; lane 3, α3; lane 4, α4; lane 5, α5. (B) Lane 1, β1; lane 2, β2; lane 3, β3. (C) Lane 1, γ1; lane 2, γ2; lane 3, γ3. The migration of marker proteins is indicated.

All 11 chain fragments were produced by recombinant expression in bacteria (Fig. 1A–C). The pPEP-T vector was used for expression because α chains in particular are unfolded when individually expressed and notoriously difficult to produce (Kammerer et al., 1995). pPEP-T, which encodes a short 6xHis-tagged C-terminal fragment of the laminin β1 chain and a thrombin cleavage site, was originally designed to express disordered laminin chains (Brandenberger et al., 1996). Some fusion proteins (α5, β1–3 and γ3) were difficult to purify due to aggregation/precipitation problems. A likely explanation for these purification difficulties is that the pPEP-T leader alone forms oligomers. These chain fragments were therefore subcloned into a different bacterial expression vector (pHis), which only provides an N-terminal 6xHis tag and a thrombin cleavage site. All chain fragments produced appreciable amounts of protein and corresponded to the expected molecular size.

### 3.2 Complex formation analysed by non-denaturing gel electrophoresis and analytical ultracentrifugation

Non-denaturing gel electrophoresis is performed in the absence of SDS or any other denaturing agents, allowing analysis of intact protein complexes that migrate according to their mass, shape and charge. The technique was used to test all 45 possible chain combinations. Complex composition of a single band was identified by in-gel trypsin digestion and subsequent mass spectrometry analysis.

**Fig. 2:**
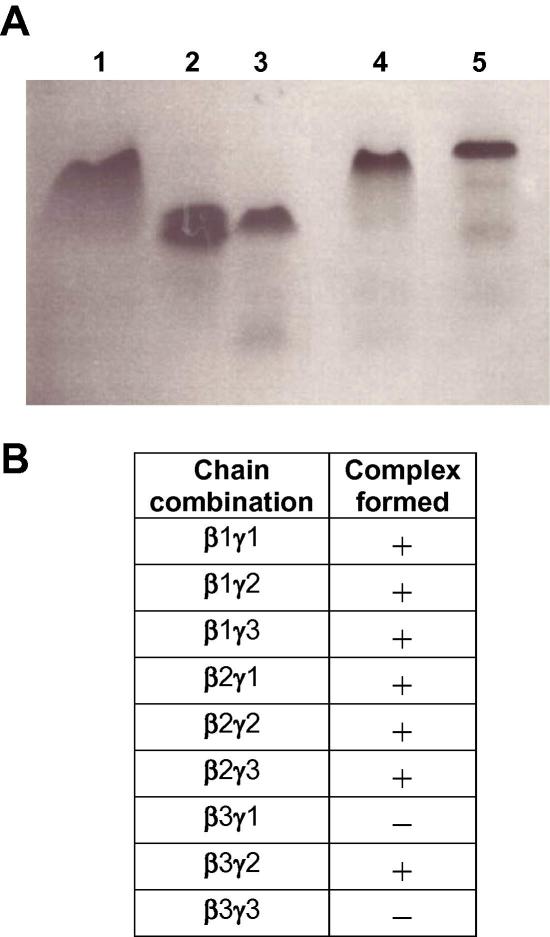
β and γ Chain complexes. (A) 10% non-denaturing gel showing heterotypic complex formation of selected chain combinations (lane 4, β1γ2; lane 5, β1γ1) in comparison with the corresponding homotypic laminin coiled-coil fragments (lane 1, β1; lane 2, γ1; lane 3, γ2). (B) Summary of heterotypic complex formation of all possible β and γ chain combinations. Complex formation on non-denaturing gels was confirmed by in-gel trypsin digestion and subsequent mass spectroscopy.

First, all possible βγ combinations were tested on non-denaturing gels (Fig. 2B) because it has previously been reported that laminin assembly is a two-step mechanism with a βγ dimer forming initially and the addition of the α chain occurring as a secondary event (Hunter et al., 1992; Kammerer et al., 1995; Nomizu et al., 1996). The finding that all combinations of β and γ chains, which are also found naturally within known heterotrimeric laminin isofoms (β1γ1, β1γ3, β2γ1, β2γ3 and β3γ2) were obtained, demonstrates the validity of our approach. Identification of β1γ2 and β2γ2 complexes represented novel βγ combinations which have not been previously seen within any laminin isoform, whereas β3γ3 and β3γ1 were the only βγ mixtures which fail to associate. Complex formation of two representative examples (β1γ1 and β1γ2) is shown in Fig. 2A.

To identify potentially novel laminin isoforms, all possible αβγ mixtures were analysed to determine which chain combinations could assemble together. The experimental methodology demonstrated again that all 15 known laminin isoform combinations identified *in vivo* were able to assemble in this *in vitro* system, except α3β2γ3 (323 or laminin 13) (Table 2). Notably, the existence of a laminin 323 isoform *in vivo* has been proposed only on the basis of the expression of laminin chains in the CNS (Libby et al., 2000). It was shown that α3, α4, α5 β2, β3, γ2 and γ3 chains are expressed in the neural retina. The authors speculated that the α3, β3 and γ2 chains would probably assemble to form laminin 332 (laminin 5), although they did not purify the isoform from retinal extracts. Their biochemical and expression data suggest the presence of at least laminin 423 (α4β2γ3 or laminin 14) and laminin 523 (α5β2γ3 laminin 15) in the CNS. Furthermore, a loss of the β3 chain in the adult outer plexiform layer was observed, which suggests that laminin 13 (α3β2γ3) could potentially exist in the CNS, although the authors were unable to demonstrate its presence biochemically.

**Fig. 3:**
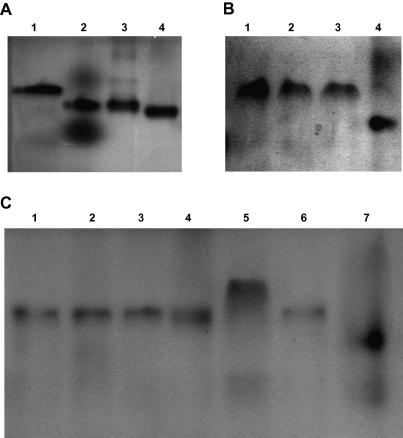
Selected laminin αβγ chain combinations. (A) 10% non-denaturing gel of selected chain combinations representing known laminin isoforms (lane 2, α1β1γ1 complex; lane 3, α2β1γ1 complex; lane 4, α3β1γ1 complex) shown in comparison to the β1γ1 complex (lane 1). α Chains are positively charged and therefore do not enter the gel. (B) 10% non-denaturing gel of αβγ chain combinations of unknown laminin isoforms (lane, 2, α1 + β1γ2; lane 3, α2 + β1γ2; lane 4, α3β1γ2) shown in comparison to the β1γ2 complex (lane 1). (C) 10% non-denaturing gel of αβγ chain combinations of unknown laminin chain combinations (lane, 2, α1 + β2γ2; lane 3, α2 + β2γ2; lane 4, α3 + β2γ2; lane 5, α4β2γ2; lane 6, α5 + β2γ2) shown in comparison to the β1γ2 complex (lane 1). Lane 7, α4 chain.

Surprisingly, only two additional heterotrimeric chain complexes were identified, α3β1γ2 (Fig. 3B) and α4β2γ2 (Fig. 3C), from the 30 remaining possible combinations. Both complexes could represent potentially novel laminin isoforms, however the subsequent biophysical analysis of α4β2γ2 was prevented due to precipitation problems during sample preparation. As a result of these technical difficulties, we therefore focused our attention on the characterisation of the novel α3β1γ2 complex. As a positive control for comparison, we used the naturally known α3β1γ1 chain combination (laminin 311, laminin 6), which differs from α3β1γ2 only by its γ chain.

Representative examples of βγ complexes that were able or failed to associate with α chains are shown in Fig. 3. Despite their positive net charge combining α1, α2 or α3 with β1γ1 all produced bands which migrated faster than β1γ1 alone and represented heterotypic associations of all three chains, as confirmed by mass spectrometry (Fig. 3A). The faster electrophoretic mobility indicates a more tight packing of the complex upon the addition of α chains. The two additional, ill-defined bands seen in lane 2 most likely correspond to small, negatively-charged degradation products of the laminin α1 chain because the full-length α1 chain fragment will not enter the non-denaturing gel as a result of its positive net charge. This conclusion is furthermore supported by the observation that these “bands” are not seen for the β1γ1 complex (Fig. 3A) or samples containing the α2 or α3 chain (Fig. 3A). In contrast, mixing β1 and γ2 chains with α1 or α2 resulted in a band that migrated at the same position as the β1γ2 complex (Fig. 3B) and as expected, no α chain was detected in the complexes upon mass spectrometry analysis. Likewise, combining β2 and γ2 chains with α1–3 and α5 yielded bands the same electrophoretic mobility as the β2γ2 dimer (Fig. 3C). Due to their positive net charge, α1, α2, α3 and α5 chains are not visible on the gel as they do not enter the gel under the conditions applied in the experimental setting. As a result of its overall negative net charge, the laminin α4 chain is the only α chain that enters the gel (Fig. 3C). In comparison, the newly identified combinations α3β1γ2 (Fig. 3B) and α4β2γ2 (Fig. 3C), however, produced predominant bands which migrated a different positions than the respective βγ complexes alone. The ill-defined additional band seen in the α3β1γ2 sample corresponds to the α3 chain as revealed by mass spectrometry. Since there is also a trace amount of uncomplexed β1γ2 detectable in the lane, the most likely explanation for the additional bands is partial dissociation of α3β1γ2 under the conditions non-denaturing gel electrophoresis was performed (room temperature, no cooling).

**Fig. 4:**
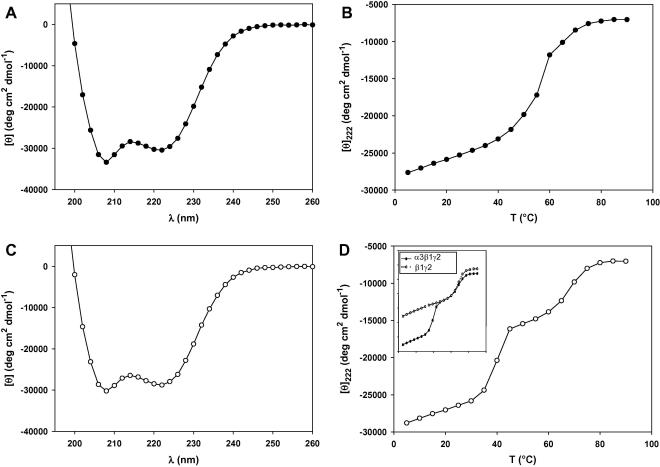
Circular Dichroism (CD) spectroscopy measurements on recombinant laminin α3β1γ1 and α3β1γ2 complexes. CD spectra of α3β1γ1 (A) and α3β1γ2 (C) recorded at 4 °C. Thermal unfolding profiles of α3β1γ1 (B) and α3β1γ2 (D) monitored by CD following temperature-induced change of the mean molar residue ellipticity at 222 nm, [Θ]_222_. Inset, qualtitative comparison of the temperature-induced unfolding of β1γ2 and α3β1γ2. All measurements were taken at total chain concentration of 10 μM. All protein samples were in 5 mM sodium phosphate buffer (pH 7.4) containing 150 mM NaCl.

To determine the subunit stoichiometry of the β1γ1, β1γ2, α3β1γ2 and α3β1γ1 heteromeric complexes, all four peptide combinations were analysed by analytical ultracentrifugation (AUC) (Table 3). Sedimentation equilibrium experiments of β1γ1 revealed a molecular mass that is in agreement with the formation of a heterodimer. In contrast, AUC revealed the β1γ2 complex to be a heterotetramer, which most likely consists of two β1 chains and two γ2 chains because equimolar mixtures of the two proteins produced only one distinct band on non-denaturing gels (Fig. 2A). For both α3β1γ2 and α3β1γ1 molecular mass values were obtained that are consistent with the formation of heterotrimeric structures consisting each of one α, β and γ chain. The structural rearrangement of the β1γ2 complex from a tetramer into a trimer upon addition of the α3 chain furthermore supports our conclusion that the heterotetramer consists of two β1 chains and two γ2 chains. Despite the presence of two additional bands on non-denaturing gels (Fig. 3B), only one molecular mass has been observed for α3β1γ2. It should be noted that AUC has been performed at 4 °C and non-denaturing gel electrophoresis at room temperature. Therefore, the most likely explanation for this discrepancy is a partial dissociation of the α3β1γ2 complex at room temperature, which is consistent with the circular dichroism (CD) results (see Fig. 4D).

Consistent with the presence of extended coiled-coil structures, sedimentation velocity experiments revealed sedimentation coefficients that were all characteristic of an elongated shape of the complexes (Table 3).

### 3.3 Structural analysis of α3β1γ2 and α3β1γ1 by circular dichroism spectroscopy and electron microscopy

CD spectroscopy was used to test for the secondary structure and stability of the heterotrimeric α3β1γ2 and α3β1γ1 laminin complexes (Fig. 4). The far-ultraviolet CD spectrum recorded at 5 °C from α3β1γ2 and α3β1γ1 was characteristic of α-helical spectra with the typical minima at 208 and 222 nm (Fig. 4A and C). Mean molar ellipticity values of about –30,000 deg cm^2^ dmol^−1^ indicated an α-helical content of 75% for both complexes. These values are consistent with a previous study in which a similarly-sized α2β1γ1 (laminin 211, laminin 2) complex from the C-terminal end of the coiled-coil domain was reported to be 75% helical (Utani et al., 1995).

Temperature-induced CD unfolding profiles were used to assess the stability of the recombinant laminin complexes (Fig. 4B and D). The thermal unfolding profile recorded from α3β1γ1 exhibited the sigmoid shape typical for coiled coils, implying a two-state transition (Fig. 4B). Accordingly, the profile was monophasic and reversible with >90% of the starting signal regained on cooling (data not shown). As expected, concentration dependence of the midpoint of thermal unfolding (*T*_m_) was observed for the mixed peptides (data not shown). At a total peptide concentration of 11 μM, the profile showed a *T*_m_ at 57 °C. In contrast, the thermal melting profile of α3β1γ2 exhibited two transitions, one with a *T*_m_ at 40 °C and the other having a *T*_m_ at 66 °C (Fig. 4D). The upper transition superimposes well to the thermal unfolding profile of the β1γ2 complex (Fig. 4D, inset), indicating that the lower transition corresponds to the unfolding of the α3 chain from the β1γ2 heteromer (Fig. 4D, inset). This observation is consistent with the previously suggested two-step mechanism of laminin assembly in which β and γ chains associate first followed by the addition of the α chain as a secondary step (Nomizu et al., 1996; Kammerer et al., 1995; Hunter et al., 1992).

**Fig. 5:**
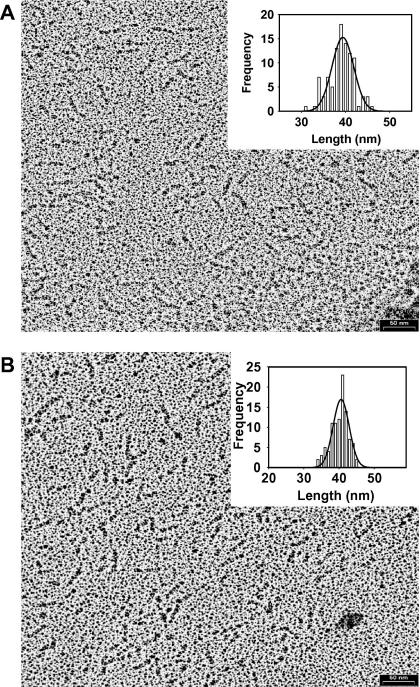
Electron microscopic analysis of recombinant laminin complexes. TEM micrographs of glycerol-sprayed/rotary metal shadowed α3β1γ1 (A) and α3β1γ2 (B) specimens. Histograms with single Gaussian fit representing the distribution of the molecular length of laminin complexes are shown as insets. One hundred molecules were measured, with values displayed representing the mean and standard deviation of the histogram. Scale bar, 50 nm.

Extended coiled-coil domains form rod-like structures that can be easily visualised by electron microscopy (Paulsson et al., 1985; Steinmetz et al., 1998). Inspection of the α3β1γ1 and α3β1γ2 complexes by transmission electron microscopy (TEM) after glycerol-spraying and rotary metal-shadowing yielded well-defined, uniformly-sized, short rod-like structures (Fig. 5). The apparent length of the molecules was measured and put into a histogram (Fig. 5, insets). A Gaussian fit of the size distribution histogram revealed a mean molecular length of 39 nm for both complexes. As both complexes correspond to approximately half of the coiled-coil domain, these values are consistent with the size of 77 nm reported for full-length laminin 111 coiled-coil domain (Engel et al., 1981). They furthermore are in good agreement with the calculated length of ∼35 nm for both coiled coils by assuming that the axial rise per residue corresponds to 1.54 A.

## 4 Conclusions

Taken together, these findings demonstrate that laminin isoform assembly is a highly specific process that is controlled by specific interactions in the C-terminus of the coiled-coil domain and not only by differential expression of laminin chains. For the first time, complex formation for all 45 possible heterotrimeric laminin chain combination was evaluated *in vitro*, which resulted in only 16 distinct laminin heterotrimeric complexes. Of these, 14 had been previously identified as laminin isoforms *in vivo* and the remaining two represent the novel potential isoforms 321 and 422. Our results demonstrate that the characterisation of isolated laminin coiled-coil domains provides an attractive approach to identify potential new isoforms within this protein family. This successful experimental strategy relies on the fact that isolated coiled-coil domains normally display the same oligomerisation state and specificity as the full-length proteins (Frank et al., 2002).

Highlighting which of the 45 possible heterotrimeric laminin chain combinations assembled *in vitro*, revealed major differences in chain specificity. Ten of the 16 isoforms identified in this study contain the γ1 chain and three isoforms each contain γ2 and γ3 (Table 2). Our results indicate that the γ chains are a significant determinant for chain specificity and selectivity. While the γ1 chain with its two possible β chain partners (β1 and β2) readily associated with all the five α chains, substituting the γ1 chain for either γ2 or γ3 dramatically altered the specificity for α chains and only resulted in six additional laminin isoforms, including the two novel potential isoforms reported in this paper. This evidence of chain selectivity provided by the γ chains suggests the existence of particular sequences within their C-terminal coiled-coil domain that determine which chains are able to associate with each other.

Whether the here characterised potential new laminin isoform 312 exists *in vivo* remains to be confirmed. Previous to this study the laminin γ2 chain was an unique subunit of laminin 332 (laminin 5) and was found to be expressed in various epithelial tissues, such as skin, lung, small intestine, stomach and kidney (Mizushima et al., 1998). Interestingly, the β1 chain also has similar expression patterns highlighting that both laminin 312 and 332 could be expressed in similar tissues supporting epithelial structures (Miner, 2008).

## Figures and Tables

**Table 1:** Recombinant mouse laminin chain fragments used in this study.

Laminin chain	Amino acid positions	Chain length	Net charge
α1	1911–2125	215	+5
α2	1924–2145	222	+5
α3	2176–2397	222	+2
α4	616–842	227	−6
α5	2525–2748	224	+9
β1	1561–1786	226	−12
β2	1575–1799	225	−3
β3	945–1168	224	+3
γ1	1362–1607	246	−14
γ2	949–1191	243	−18
γ3	1344–1581	238	+8

The chain fragments span the shown amino acid positions of the native laminin chains. The length and overall net charge of the chain fragments is indicated.

**Table 2:** Summary of non-denaturing PAGE analysis of all possible heterotrimeric laminin chain combinations.

Chain combination	Isoform	Complex formed	Chain combination	Isoform	Complex formed
*α1β1γ1*	*111/LN1*	*+*	α3β2γ2	322	−
α1β1γ2	112	−	*α3β2γ3*	*323/LN13*	*−*
α1β1γ3	113	−	α3β3γ1	331	−
*α1β2γ1*	*121/LN3*	*+*	*α3β3γ2*	*332/LN5*	*+*
α1β2γ2	122	−	α3β3γ3	333	−
α1β2γ3	123	−	*α4β1γ1*	*411/LN8*	*+*
α1β3γ1	131	−	α4β1γ2	412	−
α1β3γ2	132	−	α4β1γ3	413	−
α1β3γ3	133	−	*α4β2γ1*	*421/LN9*	*+*
*α2β1γ1*	*211/LN2*	*+*	**α4β2γ2**	**422**	**+**
α2β1γ2	212	−	*α4β2γ3*	*423/LN14*	*+*
*α2β1γ3*	*213/LN12*	*+*	α4β3γ1	431	−
*α2β2γ1*	*221/LN4*	*+*	α4β3γ2	432	−
α2β2γ2	222	−	α4β3γ3	433	−
α2β2γ3	223	−	*α5β1γ1*	*511/LN10*	*+*
α2β3γ1	231	−	α5β1γ2	512	−
α2β3γ2	232	−	α5β1γ3	513	−
α2β3γ3	233	−	*α5β2γ1*	*521/LN11*	*+*
*α3β1γ1*	*311/LN6*	*+*	α5β2γ2	522	−
**α3β1γ2**	**312**	***+***	*α5β2γ3*	*523/LN15*	*+*
α3β1γ3	313	−	α5β3γ1	531	−
*α3β2γ1*	*321/LN7*	*+*	α5β3γ2	532	−
			α5β3γ3	533	−

Complex formation was confirmed by in-gel digestion with trypsin and subsequent mass spectroscopy analysis (data not shown). Combinations forming heterotypic complexes containing all three different chains are indicated (+) and chain combinations which did not form a complex or complexes which did not contain all three different laminin chains are also indicated (−). Chain combinations corresponding to known and potentially novel laminin isoforms are highlighted in italics and bold, respectively.

**Table 3:** Molecular masses of laminin complexes determined by analytical ultracentrifugation.

Protein fragment	Sedimentation coefficient s_20w_	AUC	Calculated molar mass (kDa)
β1γ1	2.4	59	53 (1:1)
β1γ2	3.3	92	52.4 (1:1)
α3β1γ1	3.1	77	77 (1:1:1)
α3β1γ2	2.9	77	77 (1:1:1)

The recombinant proteins were analysed in 5 mM sodium phosphate buffer (pH 7.4) containing 150 mM NaCl. Calculated molecular masses of the examined heterodimers (β1γ1 and β1γ2) and hetetrotrimers (α3β1γ1 and α3β1γ2) are indicated.
